# Deep-learning model for predicting physical fitness in possible sarcopenia: analysis of the Korean physical fitness award from 2010 to 2023

**DOI:** 10.3389/fpubh.2023.1241388

**Published:** 2023-08-08

**Authors:** Jun-Hyun Bae, Ji-won Seo, Dae Young Kim

**Affiliations:** ^1^Able-Art Sport, Department of Theory, Hyupsung University, Hwaseong, Gyeonggi-do, Republic of Korea; ^2^Department of Physical Education, Seoul National University, Seoul, Republic of Korea; ^3^Senior Exercise Rehabilitation Laboratory, Department of Gerokinesiology, Kyungil University, Gyeongsan, Gyeongsangbuk-do, Republic of Korea

**Keywords:** deep learning, stratified k-fold, sarcopenia, physical fitness, aging, prediction

## Abstract

**Introduction:**

Physical fitness is regarded as a significant indicator of sarcopenia. This study aimed to develop and evaluate a deep-learning model for predicting the decline in physical fitness due to sarcopenia in individuals with potential sarcopenia.

**Methods:**

This study used the 2010–2023 Korean National Physical Fitness Award data. The data comprised exercise- and health-related measurements in Koreans aged >65 years and included body composition and physical fitness variables. Appendicular muscle mass (ASM) was calculated as ASM/height^2^ to define normal and possible sarcopenia. The deep-learning model was created with EarlyStopping and ModelCheckpoint to prevent overfitting and was evaluated using stratified k-fold cross-validation (*k* = 5). The model was trained and tested using training data and validation data from each fold. The model’s performance was assessed using a confusion matrix, receiver operating characteristic curve, and area under the curve. The average performance metrics obtained from each cross-validation were determined. For the analysis of feature importance, SHAP, permutation feature importance, and LIME were employed as model-agnostic explanation methods.

**Results:**

The deep-learning model proved effective in distinguishing from sarcopenia, with an accuracy of 87.55%, precision of 85.57%, recall of 90.34%, and F1 score of 87.89%. Waist circumference (WC, cm), absolute grip strength (kg), and body fat (BF, %) had an influence on the model output. SHAP, LIME, and permutation feature importance analyses revealed that WC and absolute grip strength were the most important variables. WC, figure-of-8 walk, BF, timed up-and-go, and sit-and-reach emerged as key factors for predicting possible sarcopenia.

**Conclusion:**

The deep-learning model showed high accuracy and recall with respect to possible sarcopenia prediction. Considering the need for the development of a more detailed and accurate sarcopenia prediction model, the study findings hold promise for enhancing sarcopenia prediction using deep learning.

## 1. Introduction

Sarcopenia is a severe health problem characterized by a reduction in muscle quality and quantity (1–4), leading to a decline in physical fitness and strength. The reduced quality of life and diminished functionality are the primary concerns associated with sarcopenia (2), which can increase societal costs and individual health concerns (5–7), thereby highlighting the importance of early prevention and treatment for sarcopenia.

A decline in physical fitness has been shown to be highly related to the incidence and mortality of sarcopenia (8). Previous studies reported that a lower level of absolute grip strength (upper strength) (9), lower level of strength on the chair sit-and-stand test (10), lower level of flexibility on the sit-and-reach test (11), lower level of cardiorespiratory endurance on the 2-min step test (12), lower level of balance on the 3-m timed up-and-go (TUG) test (13), and lower level of coordination on the figure-of-8 walk test (14) were highly associated with the diagnosis and prediction of sarcopenia. However, accurately measuring various aspects of physical fitness and understanding how they influence each other and contribute to the risk of sarcopenia remains a significant challenge in predicting sarcopenia.

Deep neural network (DNN) and machine learning (ML) algorithms have been proposed to overcome the challenges in accurately predicting sarcopenia using blood markers and skeletal muscle images (7, 11, 15–20). On the one hand, an ML model based on support vector regression, decision tree, random forest regression, or extreme gradient boosting has been used to predict physical fitness variables in older adults (15, 17, 20). On the other hand, a deep-learning-based model has been utilized to analyze computed tomography images and predict sarcopenia, and this model has also been reported to effectively predict the quality and strength of muscles in patients with cancer (21). Furthermore, a previous study developed a deep-learning-based sarcopenia prediction model (wide and deep) using clinical laboratory markers (22), which demonstrated high accuracy (area under curve [AUC] score), as compared with that of ML model prediction methods (support vector regression, random forest regression, and extreme gradient boosting). Additionally, deep learning applications in healthcare are rapidly evolving (23–25), with significant advancements in sarcopenia classification models using computed tomography (CT) (23). Several studies have used ML models to predict sarcopenia using laboratory markers and muscle mass measurements or images, without incorporating physical fitness variables (24, 26). Therefore, many subjects with sarcopenia are required to analyze and predict deep-learning models of the details of physical fitness variables.

Physical fitness is regarded as a significant indicator of sarcopenia. Nonetheless, previous studies have attempted to predict sarcopenia by measuring only the muscle quantity and blood markers without physical fitness (7, 11, 15, 18, 22). Applying a deep-learning model could provide an accurate approach to predicting sarcopenia by analyzing physical fitness and its relationships. Additionally, the accurate prediction of sarcopenia is challenging without considering physical fitness as blood markers and muscle quantity alone are insufficient indicators. Therefore, the present study aimed to develop and analyze a deep-learning model for predicting the decline in physical fitness due to sarcopenia in individuals with potential sarcopenia. This research sought to accurately predict physical fitness using a deep-learning model and to propose effective preventive and treatment strategies against sarcopenia.

## 2. Materials and methods

### 2.1. Dataset

For this type of study, formal consent was not required. The dataset was approved by the Research Ethics Committee of Hyupsung University (IRB no: 7002320-202303-HR-001), and all methods were performed in accordance with the relevant guidelines.

**Figure 1:**
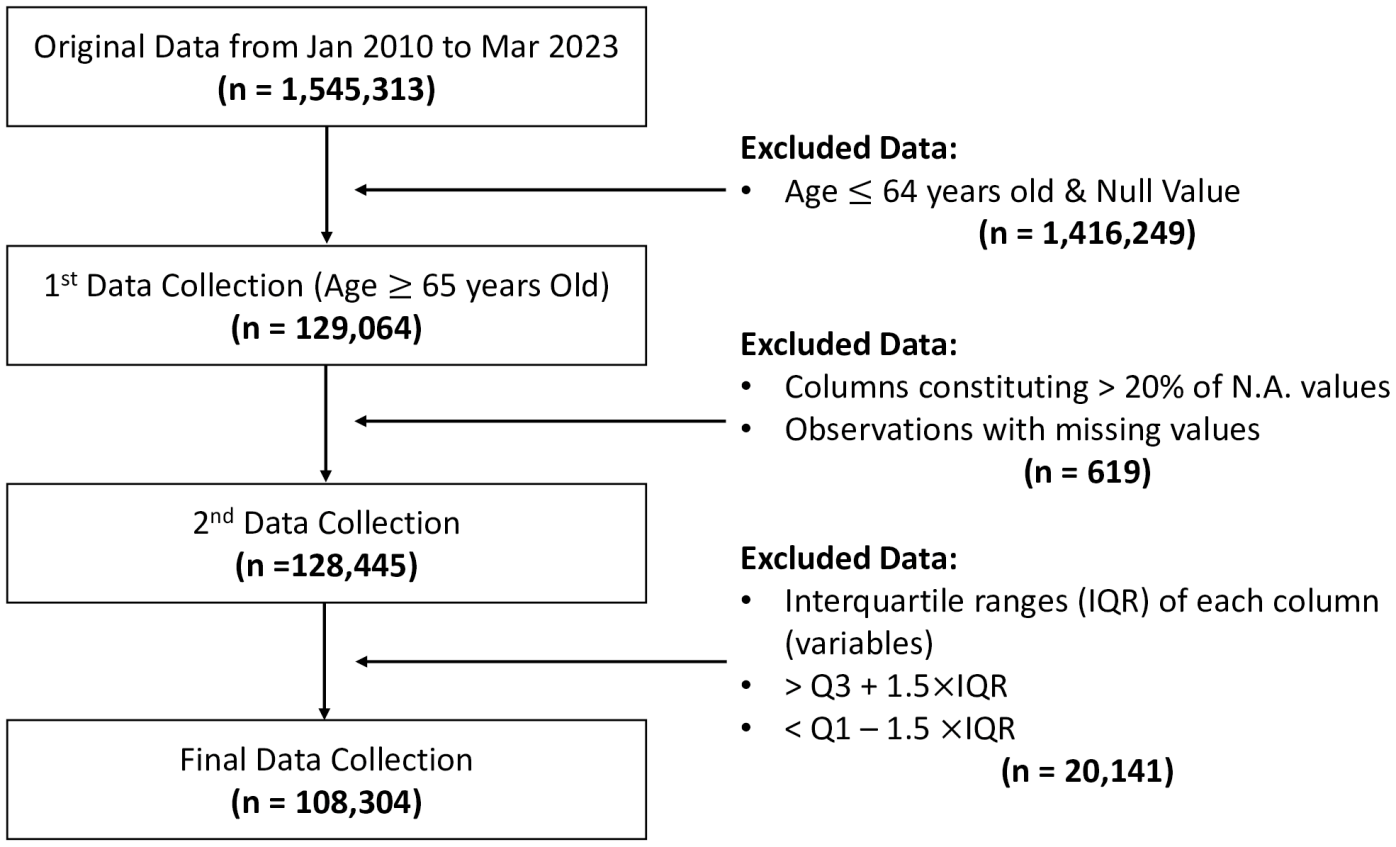
This figure indicated how the data collection in this study. From original data from January 2010 to March 2023 was 1,545,313 subjects. This study conducted excluded data following the 2.2 Data variables and data collection section. The number of final data collection was 108,304 subjects.

The present study used the 2010–2023 Korean National Physical Fitness Award data. The data comprised exercise- and health-related measurements in Koreans aged >65 years and were collected from 19 national fitness centers. The original Korean Fitness Award data were collected from Jan 2010 to Mar 2023 (*n* = 1,545,313), and the first stage excluded data from persons aged <64 years (*n* = 1,416,249). In the second stage, data of individuals with >20% missing values (*n* = 619) were excluded along with values > Q3 + 1.5*IQR or < Q1–1.5*IQR (Q, quartile; IQR, interquartile range; *n* = 20,141). The final sample size was a 108,304 participants (Figure 1). All participants voluntarily participated in the Korean National Physical Fitness Award Project through the national fitness center in each region. Body mass index (BMI, kg/m^2^), body fat (BF, %), and waist circumference (WC, cm), and physical fitness variables, such as absolute grip strength (kg), chair sit-and-stand up (counts), sit-and-reach (cm), 2-min step (counts), 3-m TUG (sec), and figure-of-8 walk (sec) (7, 11, 18), were all measured in Koreans aged >65 years. Specific measurements were conducted by trained physical fitness instructors (27) and were performed on the basis of the Survey of National Physical Fitness (7, 11, 18) and Development of National Physical Fitness Certification Standards for older adults (7, 11, 18). The analysis environment was Apple M1 Max with macOS Ventura 13.4, 32 GB RAM, and NVIDIA A100-SXM4-40GB. The analysis program was Google Colaboratory (Colab) with a cloud-based platform that offered high GPU for computing purposes, which was based on Python 3.10.11 (28).

### 2.2. Data variables and data collection

In this study, the appendicular muscle mass (ASM, kg) was quantified and estimated using high-quality anthropometric formulas (4, 6, 29). The ASM (*R*^2^ = 0.90, standard error = 1.35 kg) was calculated as 0.193*Weight (kg) + 0.107*Height (cm) – 4.157*sex (1 for male, 2 for female) – 0.037*Age (years) – 2.631 (29). ASM/ht^2^ was calculated as a measure of ASM adjusted for the square of height in meters, and the ASM/ht^2^ value of the 20th percentile was used to define low muscle mass, similar to previous studies (1–3). In this study, low muscle mass was defined as an ASM/ht^2^ value of <6.54 for men and <5.14 for women. The 20th percentile was subsequently divided into two categories: normal (*n* = 103,546) and potential sarcopenia (*n* = 5,357). The binary dependent (normal vs. possible sarcopenia) variable was predicted using independent variables, including BF (%), WC (cm), and physical fitness measures such as sit-and-stand up (counts), 2-min step (counts), TUG (sec), figure-of-8 walk (sec), absolute grip strength (kg), and sit-and-reach (cm). Table 1 summarizes all variables of this dataset between normal and possible sarcopenia, whereas Figure 1 presents how the data were collected.

**Table 1:** The results of differences between normal and possible sarcopenia.

Variables	Normal (*n* = 102,919)	Possible sarcopenia (*n* = 5,385)	*t*	*p*-value
Age (years)	71.37 ± 4.77	74.18 ± 5.58	−41.67	0.000
Gender (M = 1/F = 2)	1.57 ± 0.50	1.90 ± 0.30	−48.04	0.000
ASM/ht^2^ (kg/m^2^)	6.75 ± 0.98	5.04 ± 0.50	126.59	0.000
Height (cm)	158.58 ± 8.13	151.75 ± 7.14	60.44	0.000
Weight (kg)	62.23 ± 8.39	45.27 ± 3.25	147.76	0.000
Waist circumference (cm)	84.67 ± 7.38	72.80 ± 5.12	116.61	0.000
Absolute grip strength (kg)	25.49 ± 7.79	18.98 ± 5.01	60.69	0.000
Sit and reach (cm)	10.63 ± 8.77	12.14 ± 7.97	−12.38	0.000
Body fat (%)	31.01 ± 7.40	25.80 ± 6.25	50.71	0.000
BMI (kg/m^2^)	24.73 ± 2.60	19.72 ± 1.61	140.04	0.000
Sit and stand (count)	20.70 ± 5.82	19.46 ± 5.71	15.26	0.000
2-min step (count)	110.43 ± 17.59	105.02 ± 19.23	21.89	0.000
TUG (sec)	6.01 ± 1.11	6.40 ± 1.24	−24.95	0.000
Fig-8 walk (sec)	24.60 ± 4.77	26.11 ± 5.24	−22.63	0.000

This table described the results of differences between normal (*n* = 102,919) and possible sarcopenia (*n* = 5,385). All statistical analysis was based on an independent *t*-test and the statistical α < 0.05. ASM/ht^2^, Appendicular skeletal muscle/square of height; BMI, Body mass index; 2-min step, 2 min step test; TUG, 3-m up-and-go test; Fig-8 walk, Figure-of-8 walk test.

### 2.3. Statistical modeling

#### 2.3.1. Data normalization and sampling

The data were normalized using MinMaxScaler to avoid overreliance on certain features while speed learning by restricting all variables to a range between 0 and 1. Datasets were also balanced via under-sampling using RandomUnderSampler by reducing oversampling between “possible sarcopenia” and “normal.”

### 2.4. Data analysis

#### 2.4.1. Stratified k-fold cross-validation

In this study, the dataset was divided while maintaining the same class ratio, which is particularly effective for imbalanced datasets. Five equal-size groups (*k* = 5) were used, and the data were randomly shuffled; hence, each model underwent five independent training–evaluation processes to ensure the reproducibility and reliability of the data results. Additionally, the performance metrics obtained from these processes were averaged to estimate the final performance of the model (30–32).

#### 2.4.2. Model structure and compilation

In this study, a neural network model with four layers was created. The initial layer consisted of 64 nodes; for the initial layer, the rectified linear unit (ReLU) activation function was implemented as its activation function. The input data were organized as an eight-dimensional vector to accommodate for datasets with eight independent variables. A dropout layer was employed to prevent overfitting; as part of its learning process, this layer randomly deactivated 20% of nodes within this layer during each iteration to ensure that the model did not overrely on specific nodes and to help increase the generalization capability. The third layer comprised 32 nodes using the ReLU activation function. Finally, an individual node layer was equipped with the sigmoid function to produce probabilities between 0 (“normal”) and 1 (“possible sarcopenia”), catering specifically to binary classification problems. At the compilation stage, binary cross-entropy was utilized as the loss function, providing an appropriate measure for binary classification problems by quantifying differences between predicted and actual values of models. Adam optimizer was selected as the optimization algorithm owing to its adaptive learning rate adjustment that could enhance the learning speed and overall performance. Accuracy was selected as the performance metric for evaluating the precision of classification predictions. Subsequently, this model was employed to learn from training data at each step, followed by validation data performance evaluation. This process was iterated five times using a five-fold cross-validation methodology (5, 33).

#### 2.4.3. EarlyStopping and ModelCheckpoint

EarlyStopping monitors validation loss and halts the training when validation loss does not improve after a certain number of epochs (in this study, a patience parameter = 20). This strategy prevents overfitting because it stops the training when the performance of the validation set starts to degrade. Moreover, the weights of the model at its peak performance are restored by setting the best weight, thereby ensuring the retention of the best model instead of the final one when the training has ceased. ModelCheckpoint also monitors validation loss, with the model being saved at each time when validation loss decreases during training. This strategy preserves the best performing model after the training has been completed (34, 35).

#### 2.4.4. Model training and test

Training data from each fold, as well as validation data, were used to train a model (80% of training data, 20% of validation data). The training processes were 200 epochs in length, with 16 batches of data per training run. During training, EarlyStopping and ModelCheckpoint calls were employed to monitor validation loss during learning sessions. Once loaded onto the validation data, it was subsequently predicted using this model. Results were outputted as probabilities prior to binary classification using a threshold of 0.5% (36).

#### 2.4.5. Model prediction and performance evaluation

The model’s performance was visually evaluated using a confusion matrix. Receiver operating characteristic (ROC) curves were constructed, and the AUCs were calculated. The average performance metrics obtained from each cross-validation were determined and outputted. Changes in the model’s performance were visualized on a graph showing accuracy, precision, recall, and F1 scores obtained from cross-validation (37, 38).

#### 2.4.6. Model interpretability and explanation with SHAP, permutation feature importance, and LIME

After assessing the model’s performance, SHapley Additive exPlanations (SHAP) (39), permutation feature importance (40), and Local Interpretable Model-Agnostic Explanations (LIME) (41) were employed as model-agnostic explanation methods for evaluating how the model’s prediction worked, which provided insights into which features contributed the most toward making predictions to enhance the transparency and accuracy within models.

## 3. Results

### 3.1. Results for all variables between normal and possible sarcopenia

All variables showed statistically significant differences between normal (*n* = 102,919) and possible sarcopenia (*n* = 5,385). For the results, a negative t-statistic value suggested that the mean value was higher in possible sarcopenia than in normal. Statistically negative values were obtained for age (*t* = −41.67), gender (−48.04), sit-and-reach (*t* = −12.38), TUG (*t* = −24.95), and figure-of-8 walk (*t* = −22.63), indicating that these variables were higher in possible sarcopenia (Table 1).

### 3.2. Results for multicollinearity using Pearson’s correlation, variance inflation factor, and tolerance

**Figure 2:**
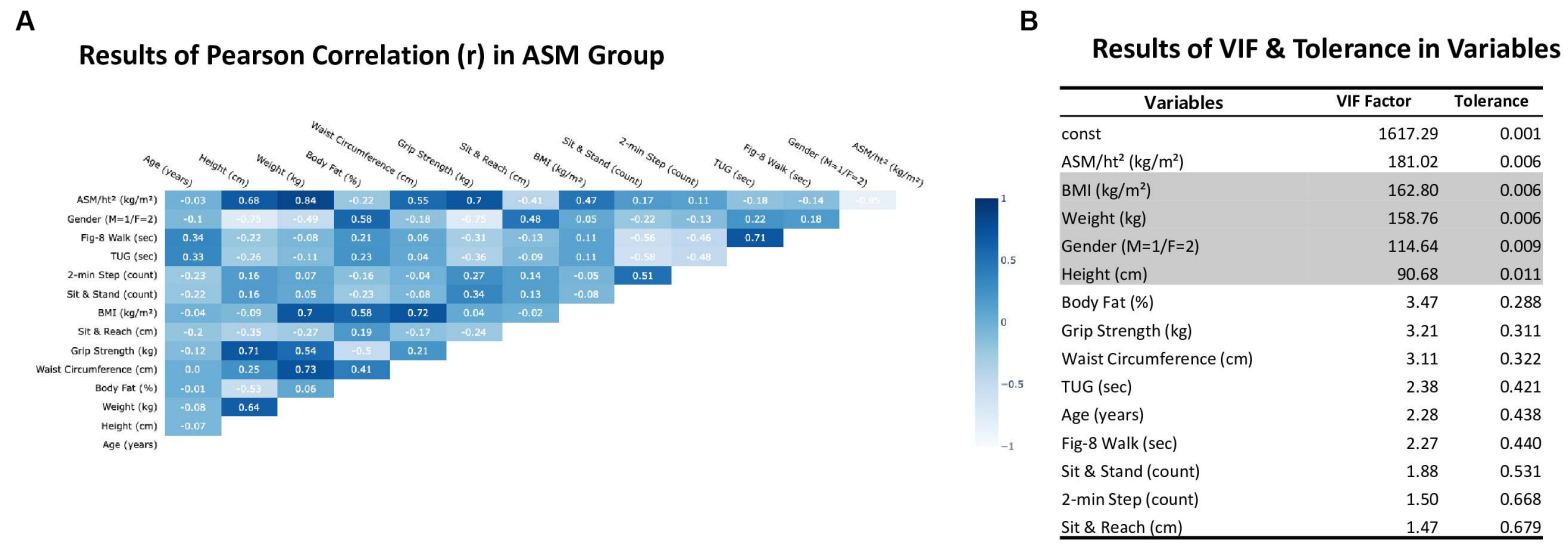
**(A,B)** described the multicollinearity in *Pearson* correlation, variance inflation factor (VIF), and tolerance. The *Pearson* Correlation (*r*) had a threshold over absolute 0.70, and then VIF threshold ≥5 and Tolerance ≤0.01 had multi-collinearity, and then remove the related features. A high density of blue color mean highly correlated in individual variables. const, constant of VIF and tolerance; ASM/ht2, Appendicular skeletal muscle/square of height; BMI, Body mass index; 2-min Step, 2 min step test; TUG, 3-m up-and-go test; Fig-8 Walk, Figure-of-8 walk test.

In this study, Pearson’s correlation (*r*) threshold >0.70, VIF threshold ≥5, and tolerance threshold ≤0.01 indicated multicollinearity; related features were subsequently removed. Pearson’s correlation coefficient of ASM/ht^2^ was >0.70 for weight and gender (Figure 2). The VIF and tolerance values for weight (VIF = 158.76, tolerance = 0.006) and gender (VIF = 114.64, tolerance = 0.009) were > 5 and < 0.1, respectively. Similarly, VIF and tolerance values for BMI (VIF = 162.80, tolerance = 0.006) and height (VIF = 90.68, tolerance = 0.011) were >5 and <0.1, respectively (Figure 2B). Pearson’s correlation coefficient for the absolute grip strength was 0.70; however, the VIF and tolerance values were 3.21 and 0.311, respectively, suggesting that the absolute grip strength did not exhibit multicollinearity. Hence, in this study, weight, height, BMI, and gender were excluded as variables due to multicollinearity, and ASM was practically quantified using an anthropometric equation based on gender, age, weight, and height. Age was excluded from the deep-learning model. Therefore, this study included independent variables, including BF (%); WC (cm); and physical fitness measures such as sit-and-stand up (counts), 2-min step (counts), TUG (sec), figure-of-8 walk (sec), absolute grip strength (kg), and sit-and-reach (cm).

### 3.3. Results for the confusion matrix from the stratified k-fold cross-validation

**Figure 3:**
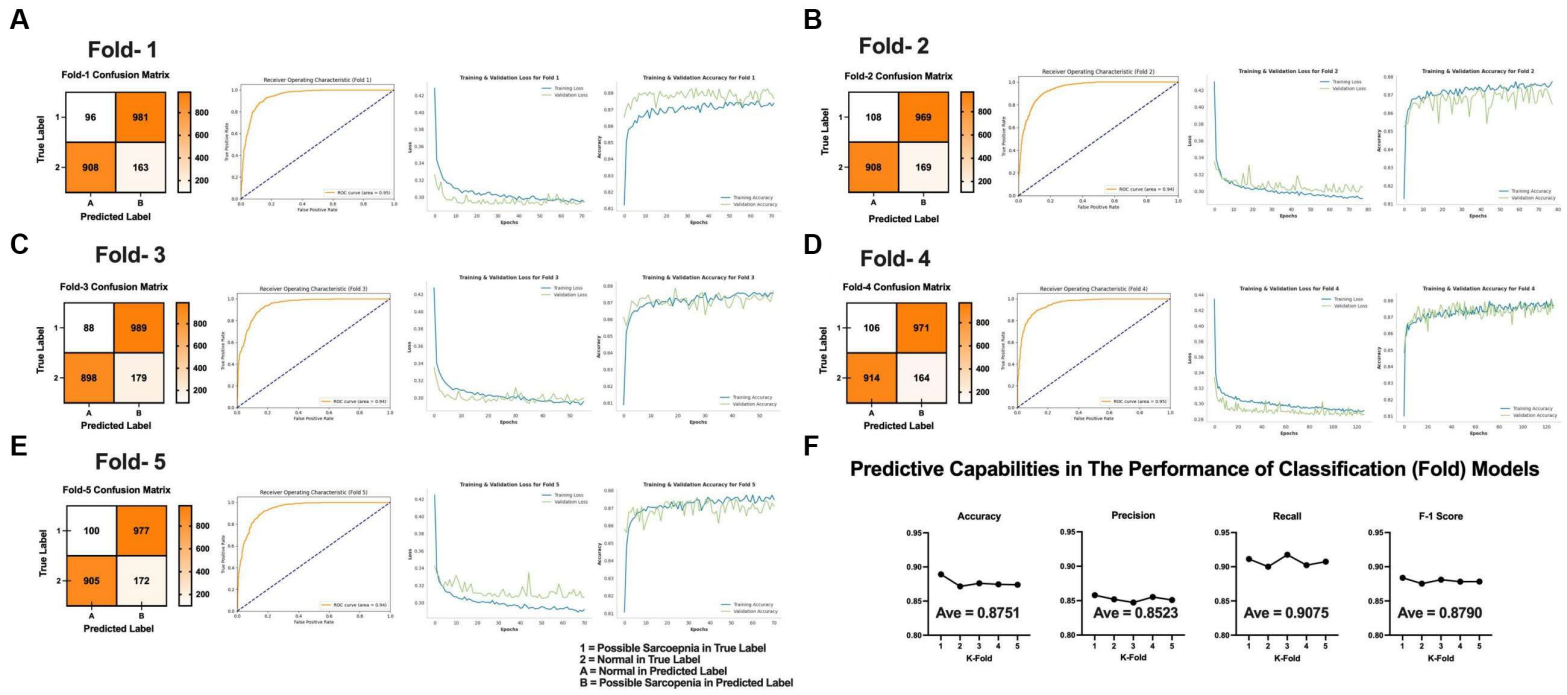
This figure showed the confusion matrix, AUC curve, and visualizing loss and accuracy for training and validation data in each fold. ROC-AUC, Receiver Operating Characteristic Curve—Area Under Curve. **(A)** Fold-1. **(B)** Fold-2. **(C)** Fold-3. **(D)** Fold-4. **(E)** Fold-5. **(F)** Predictive capabilities in the performance of classification (fold) models.

Fold-1 had early stopping in 72 epochs (ROC-AUC, 0.95), with a train loss value of 0.2940, train accuracy of 0.8738, validation loss value of 0.2950, and validation accuracy of 0.8765 (Figure 3A). Fold-2 had early stopping in 78 epochs (ROC-AUC, 0.94), with a train loss value of 0.2919, train accuracy of 0.8773, validation loss value of 0.3048, and validation accuracy of 0.8649 (Figure 3B). Fold-3 had early stopping in 56 epochs (ROC-AUC, 0.94), with a train loss value of 0.2957, train accuracy of 0.8755, validation loss value of 0.2997, and validation accuracy of 0.8770 (Figure 3C). Fold-4 had early stopping in 127 epochs (ROC-AUC, 0.95), with a train loss value of 0.2914, train accuracy of 0.8766, validation loss value of 0.2853, and validation accuracy of 0.8760 (Figure 3D). Finally, Fold-5 had early stopping in 71 epochs (ROC-AUC, 0.94), with a train loss value of 0.2921, train accuracy of 0.8747, validation loss value of 0.3065, and validation accuracy of 0.8709 (Figure 3E). The mean squared error from each fold was 0.0911, the mean average error was 0.1813, and average ROC-AUC was 0.9445 (Figure 3F).

### 3.4. Results for the deep-learning model from the stratified k-fold cross-validation

As shown in Figure 3F, the deep-learning model was trained and evaluated on our dataset using stratified k-fold cross-validation (*k* = 5). Biases in training and validation data were minimized by partitioning the data into training and verification sets while preserving the overall distribution patterns. “Normal” and “possible sarcopenia” were differentiated using “0” and “1” as indicators of normality. Our performance metrics indicated that the model exhibited an accuracy of 0.8751, implying that the model made correct predictions in 87.51% of all cases and that the model accurately classified “normal” and “possible sarcopenia.” The precision score was 0.8523, indicating a high degree of precision in prediction for the positive class (“possible sarcopenia”). In other words, 85.23% of the instances predicted as “possible sarcopenia” were indeed correctly identified. The recall score for the model was 0.9075, highlighting the model’s ability to accurately identify a high proportion of actual positive cases. That is, the model was able to correctly classify 90.75% of all actual cases of “possible sarcopenia.”

The F1-score takes both precision and recall into account and is, thus, a useful metric for evaluating the balance between the two, with a high F1-score indicating good model performance. The F1-score for our model, which is the harmonic mean of precision and recall, was 0.8790. As our model showed a high score of 87.9%, this model therefore exhibited overall high performance in classifying between “normal” and “possible sarcopenia.”

### 3.5. Results for SHAP, LIME, and permutation feature importance

**Figure 4:**
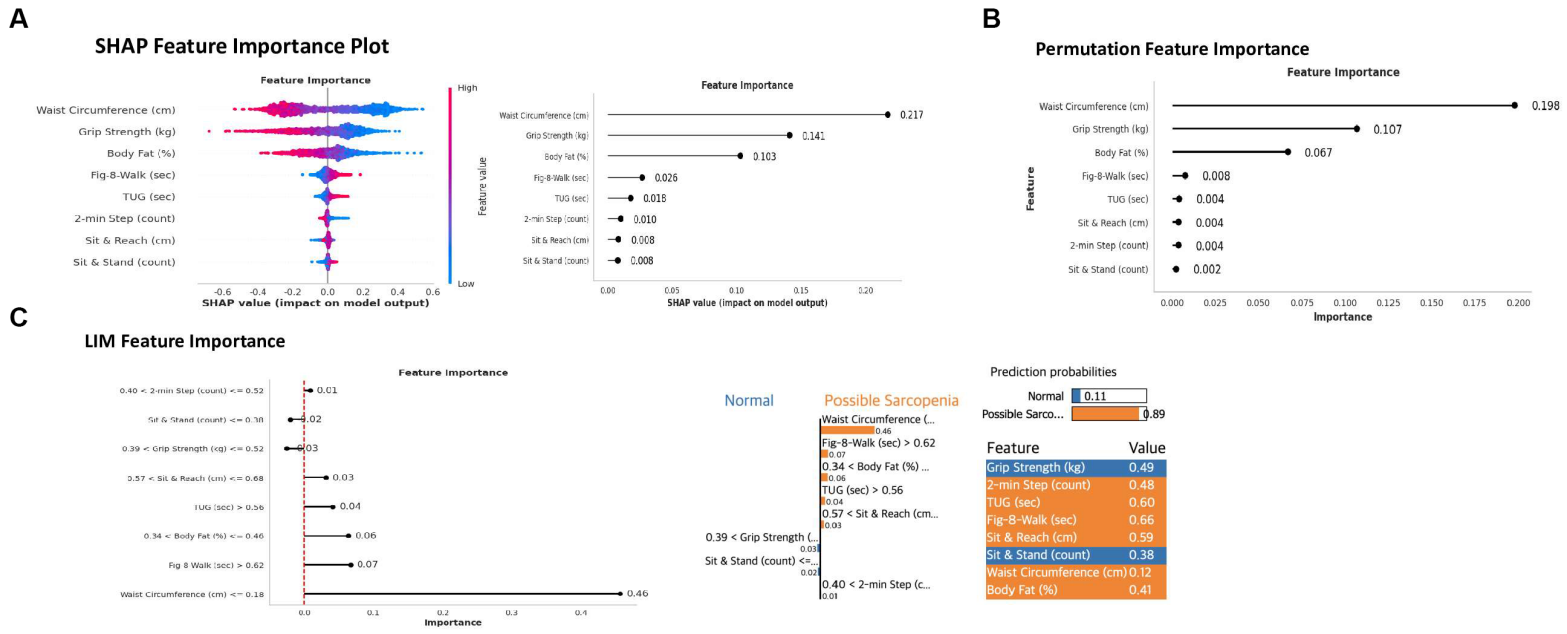
This figure showed the model-agnostic explainable algorithms from the deep learning model. The **(A)** explained feature importance from Shapley Additive exPlanations (SHAP) in the best model. The blue color mean low level impact on the model, and red coler mean high level impact on the model. The **(B)** explained permutation feature importance in the best model. The **(C)** explained Local Interpretable Model-Agnostic Explanations (LIME) feature importance in the best model and used HyperText Markup Language (HTML). Grip strength, Absolute grip strength; 2-min Step, 2 min step test; TUG, 3-m up-and-go test; Fig-8 Walk, Figure-of-8 walk test.

The SHAP results indicated that WC, absolute grip strength, and BF had a high impact on model output (Figures 4A,B). Small WC, low absolute grip strength level, and low BF level were more likely to be indicative of possible sarcopenia [Figure 4A (left), with low values for features in blue color]. Specific SHAP feature importance values are presented in Figure 4A (right); WC showed the highest importance, with a SHAP value and prediction value of 0.2170 and 0.8046, respectively. These results suggested that WC had the greatest impact on prediction. The second most important variable was absolute grip strength, with an importance value and prediction value of 0.1408 and 0.9845, respectively. The third most important variable was BF (%), with an importance value and prediction value of 0.1027 and 0.2690, respectively. The SHAP values and prediction values of the remaining variables were as follows: figure-of-8 walk (importance: 0.0265, prediction: 0.0002), TUG (importance: 0.0176, prediction: 0.0655), 2-min step (importance: 0.0100, prediction: 0.8836), sit-and-reach (importance: 0.0082, prediction: 0.9794), and sit-and-stand (importance: 0.0078, prediction: 0.1620).

The permutation feature importance results indicated that WC exhibited the highest importance in permutations, with an importance score of 0,1979 (standard deviation [SD]: 0.0048) (Figure 4B), suggesting that it had a significant impact on model predictions. The second most important variable was absolute grip strength, with an importance score of 0.1067 (SD: 0.0075), whereas the third most important variable was BF, with an importance score of 0.0668 (SD: 0.0070). The permutation importance values of the remaining variables were as follows: 0.0075 for figure-of-8 walk (SD: 0.0020), 0.0040 for TUG (SD: 0.0017), 0.0038 for sit-and-reach (SD: 0.0015), 0.0037 for 2-min step (SD: 0.0024), and 0.0024 for sit-and-stand (SD: 0.0020).

The LIME results revealed that WC (cm, 0.46) contributed the most to the prediction of possible sarcopenia (Figure 4C). A smaller WC exerted the greatest influence on prediction, followed by the figure-of-8 walk (sec, 0.07), BF (%, 0.06), TUG (sec, 0.04), and sit-and-reach (sec, 0.03). These three variables contributed more to prediction, as their values increased with possible sarcopenia. Conversely, absolute grip strength (kg, 0.03) and sit-and-stand (counts, 0.01) contributed less to prediction because as the values of these variables increased, the prediction value (normal) decreased. Finally, sit-and-reach (cm) was shown to make a weak positive contribution to prediction within a certain range.

The prediction value of probabilities was 0.89 in possible sarcopenia after LIME feature importance [Figure 4C (right)]. The variables related to “possible sarcopenia” prediction in the model were WC (cm), figure-of-8 walk (sec), BF (%), TUG (sec), and sit-and-reach (cm). The WC of values less than 0.18, which mean original values using MinMaxScaler, was 71.49 cm. Its value mean less than 71.49 cm of WC values to be a possible sarcopenia. The figure-of-8 walk (sec) of values were over 0.62, which mean 28.45 s in original value to be a possible sarcopenia. The range of BF (%) were over 0.34 and less than 0.46, which mean over 23.96% and less than 29.16% of BF to be a possible sarcopenia. The TUG also had over 0.56, which mean 6.70 s in original value to be a possible sarcopenia. The sit-and-reach (cm) had range over 0.57 to less than 0.68, which revealed 13.91–19.36 cm to be a possible sarcopenia.

## 4. Discussion

The present study used a deep-learning model to predict sarcopenia and evaluated the performance of this model using stratified k-fold cross-validation (Figure 3). The average accuracy, precision, recall, and F-1 score of our model were 87.51, 85.23, 90.75, and 87.90%, respectively, suggesting that this model can accurately distinguish normal from sarcopenia cases. In this study, we employed SHAP, LIME, and permutation feature importance methods to analyze feature importance. Through this, we found that WC (cm), absolute grip strength (kg), and BF (%) had the greatest impact on possible sarcopenia prediction, indicating that WC, absolute grip strength, and BF play a significant role in predicting possible sarcopenia and may be used to assess sarcopenia. Notably, WC emerged as the most important variable in predicting possible sarcopenia. These results can be supported deep-learning based model had diagnostic sarcopenia (22).

Our study used ASM/ht^2^ <6.54 (kg/m^2^) for men and <5.14 (kg/m^2^) for women, which supported a similar pattern of cut-off values by the Asian Working Group for Sarcopenia (3). While an anthropometric equation was used in this study to estimate ASM, the results showed a similar pattern to the findings of previous studies using the criteria cut-off value (3, 4, 29). The results also supported that a DNN based on CT-based skeletal muscle measurement was highly related to sarcopenia prediction (14, 23, 25). Based on the previously established formula of ASM (29), this study found that the accuracy of the deep-learning model in predicting sarcopenia was higher when using ASM/ht^2^, thus, supporting the potential of using physical fitness measures to predict sarcopenia.

In a previous study on data from the Korea National Health and Nutrition Examination Survey (KNHANES) conducted from 2008 to 2011, the dataset also suggested that the DNN had a significant impact on physical activity, BMI, and WC using SHAP analysis in the sarcopenia prediction model (26). The SHAP feature importance (Accuracy 84%) with the DNN model showed that WC and BMI had the highest impact on the DNN prediction model with physical activity level in daily life (26). Our results also indicated that WC and absolute grip strength were the most important features in predicting possible sarcopenia, which is a similar pattern of results that are able to explain the higher accuracy of the deep-learning model compared to that of the ML method.

Moreover, the same dataset of a previous study using Korean National Fitness Award from 2015 to 2019 indicated that DNN model represented the best performance among physical fitness variables (15–17). The study explained that including the grip strength variable as a marker of physical fitness improved the prediction of the DNN (Accuracy: 78.4%). Our deep-learning model revealed that absolute grip strength was the key variable factor in predicting possible sarcopenia (Accuracy: 87.55%); the accuracy improved by 9.15% in our study because of early stopping and using the model checkpoint method, which improved model performance and efficiency (42, 43).

Similar to previous study, our study used the same dataset and our deep-learning model was more valid through under-sampling method and stratified k-fold analysis (5, 31, 32, 44). The results supported our results, which revealed that WC (cm) and absolute grip strength had a high impact on the DNN model with SHAP, LIME, and permutation analysis (Figure 4). Moreover, WC prediction using models based on extreme gradient boosting was significantly important for epidemiology (6, 44); hence, our results suggested more details of WC with physical fitness and were similar to those of previous studies. When compared with our previous study, the result indicated that ML with CatBoost Regressor showed a good prediction of grip strength in older adults [Mean Squared Error (MSE) = 16.659]; among the seven ML models tested, it achieved the highest accuracy. However, in this study, the deep-learning model using stratified k-fold validation outperformed all others with the lowest MSE value of 0.0911. This result substantiated the superiority of the deep-learning approach over the ML approach in terms of accuracy (45).

Our study indicated that WC had a high impact on possible sarcopenia prediction (Figure 4). This result supported that sarcopenia was related to metabolic syndrome in men with normal WC and women with high WC and was predicted by abdominal obesity (46). Our SHAP and permutation feature importance analysis results also supported that WC contributed to the risk of sarcopenia with metabolic syndrome (46). The LIME analysis showed that WC had a value of less than 0.18 (original value = 71.49 cm) and the BF value ranging from 0.34 to 0.46 (original value = 23.96–29.16%) was related to possible sarcopenia. This result suggested that high WC and BF were significantly related to a lower incidence of sarcopenia (47). Moreover, the sarcopenia classification from an anthropometric method showed that WC was useful in screening for possible sarcopenia (48). This result supported our study, which considered the strong association of WC with the anthropometric method to predict possible sarcopenia. A previous study, who were in sarcopenia defined by the Asian Working Group for Sarcopenia (AWGS), showed only women with high WC and BF group had a lower incidence of sarcopenia (47). Our study also indicated that lower levels of WC and BF highly predicted possible sarcopenia (Figure 4A). When compared to the previous study, our study excluded gender variable in the results of multicollinearity (Figure 2), and the results of WC and BF from our study would change the importance factor within the gender difference. Furthermore, the deep-learning-based regression was useful for predicting grip strength in the upper strength by reducing the risk of musculoskeletal disorders (49). Our SHAP and permutation feature analysis results also supported that grip strength was the second most important variable for predicting possible sarcopenia. In addition, our deep neural prediction model had predicted that absolute grip strength had a high impact on predicting a possible sarcopenia (50). Grip strength was a valid and easy tool for early screening of sarcopenia (15–17, 51) and was highly related to physical fitness variables (15–17). Our study also demonstrated that the LIME analysis, as shown in Figure 4, indicated that the absolute grip strength ranging from 0.39 to 0.52 (original value = 19.71–25.68 kg) was associated with the normal group. Table 1 described the absolute grip strength in the possible sarcopenia group as 18.89 kg. A previous study described that grip strength (kg) was more diagnostic of sarcopenia than the chair stand test (count) (52). Our results are consistent with this, showing grip strength to be the second most important variable for predicting possible sarcopenia, in comparison to other physical fitness variables. However, according to AWGS guidelines, the grip strength for diagnosing sarcopenia was less than 28.0 kg for men and less than 17.7 kg for women (53). Our results would consider the gender difference in absolute grip strength, the importance of grip strength in the AWGS criteria would change.

The present study has some limitations. First, the defined possible sarcopenia in this study based only on the anthropometric formulas (muscle mass only) without considering muscular strength and physical function. This fundamentally differs from the diagnostic criteria by AWGS, which considers muscular strength and physical function with muscle mass. Our findings may not fully reflect the broader aspects of sarcopenia as defined by the AWGS criteria. Therefore, this limitation should be considered when interpreting and applying the results of our study. Further studies are required to analyze direct measurements of ASM/ht^2^ with physical fitness. Second, future research may incorporate additional variables, such as physical activity level and nutritional status, to improve the accuracy of the estimation results. Third, the deep-learning model used in this study showed relatively high accuracy, precision, recall, and F1 scores; however, these results do not preclude the possibility of model overfitting. While various cross-validation techniques were employed to mitigate this issue, such techniques cannot always completely prevent overfitting. Lastly, the results of this study indicated that WC, absolute grip strength, and BF play an important role in predicting sarcopenia. However, the measurement of these variables typically involves a complex process that requires professional training, which may limit their practicality in diagnosing and managing sarcopenia. Further research based on the results of this study is required to identify other variables that are easier to measure but can still provide meaningful information.

In conclusion, the results from stratified k-fold cross-validation indicated that our model exhibited high performance, with an average accuracy of 87.55%, precision of 85.57%, recall of 90.34%, and F1 score of 87.89%. These results suggest that this model can accurately classify the majority of normal and possible sarcopenia cases. Additionally, the SHAP, LIME, and permutation feature importance analysis revealed that WC, absolute grip strength, and BF had the greatest impact on model prediction for possible sarcopenia. WC, in particular, was deemed to be the most important variable. The deep-learning model exhibited high accuracy and recall in sarcopenia prediction, holding promise for enhancing sarcopenia prediction using deep learning. Nonetheless, there remains a need for the development of a more detailed and accurate sarcopenia prediction model. This would provide important insights for the prediction and management of sarcopenia and can be used in future research in this field.

## Data availability statement

The datasets presented in this study can be found in online repositories. The names of the repository/repositories and accession number(s) can be found in the article/Supplementary material.

## Ethics statement

The studies involving humans were approved by the Research Ethics Committee of Hyupsung University (IRB no.: 7002320-202303-HR-001), and all methods were performed in accordance with the relevant guidelines. The studies were conducted in accordance with the local legislation and institutional requirements. Written informed consent for participation was not required from the participants or the participants’ legal guardians/next of kin in accordance with the national legislation and institutional requirements.

## Author contributions

J-HB and DK contributed to data collection, data analysis, and writing of the manuscript. J-HB, J-wS, and DK were involved in data collection and reviewed the manuscript. All authors contributed to the article and approved the submitted version.

## Funding

This work was supported by the Kyungil University research fund.

## Conflict of interest

The authors declare that the research was conducted in the absence of any commercial or financial relationships that could be construed as a potential conflict of interest.

## Publisher’s note

All claims expressed in this article are solely those of the authors and do not necessarily represent those of their affiliated organizations, or those of the publisher, the editors and the reviewers. Any product that may be evaluated in this article, or claim that may be made by its manufacturer, is not guaranteed or endorsed by the publisher.
