# Supplementary material for: Deep-learning model for predicting physical fitness in possible sarcopenia: analysis of the Korean physical fitness award from 2010 to 2023
Source: Front Public Health. 2023 Aug 8;11:1241388. doi: 10.3389/fpubh.2023.1241388 (PMC10443707; doi:10.3389/fpubh.2023.1241388)
Supplement: Supplementary file 1 [file Data_Sheet_1.pdf]

Supplementary Material

Deep-learning model for predicting physical fitness in possible sarcopenia: analysis of the Korean Physical Fitness Award from 2010 to 2023

Jun-Hyun Bae[1], Ji-won Seo[2], Dae Young Kim[3*]

*** Correspondence:** Dae Young Kim: daeyoung@kiu.ac.kr

1 Supplementary Figures

1.1 Supplementary Figure

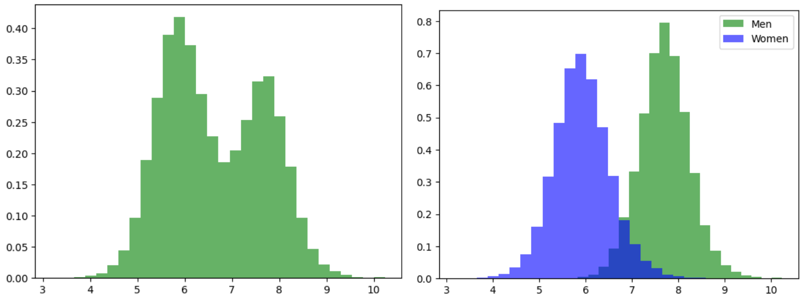

Supplementary Figure 1. This supplementary figure described the results of histogram in ASM/ht^2 in the dataset. Left graph indicated the histogram of ASM/ht^2 without separating gender. Right graph indicated the results of histogram in ASM/ht^2 dataset between men and women (<20% cut-off)

1.2 Supplementary Figure

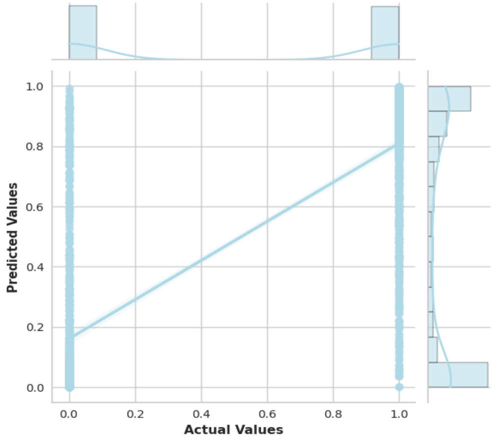

Supplementary Figure 2. This supplementary figure described the results of different residuals between predicted and actual values in the dataset.

1.3 Supplementary Figure

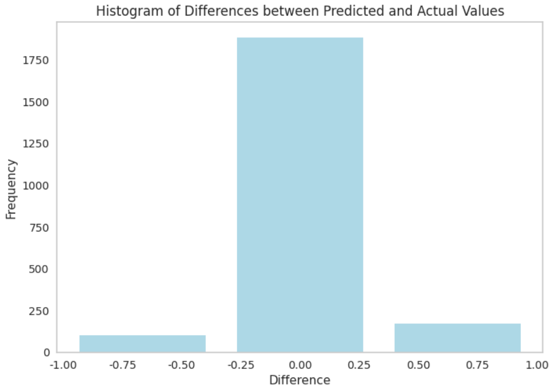

Supplementary Figure 3. This supplementary figure described the results of histogram of differences between predicted and actual values in the dataset.

1.4 Supplementary Figure

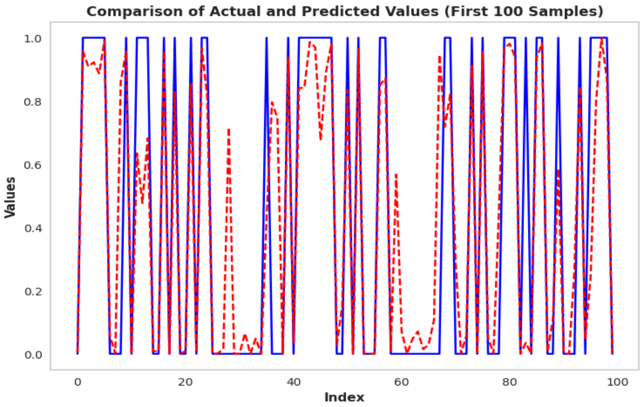

Supplementary Figure 4. This supplementary figure described the results of comparison of actual and predicted values in the first 100 samples with the best-model.
